# VDJ gene usage among B-cell receptors in ABO-incompatible kidney transplantation determined by RNA-seq Transcriptomic analysis

**DOI:** 10.1186/s12882-017-0770-8

**Published:** 2017-11-28

**Authors:** Hee Jung Jeon, Kwangsoo Kim, Jae-Ghi Lee, Joon Young Jang, Seongmin Choi, Taishi Fang, Ji-Jing Yan, Miyeun Han, Jong Cheol Jeong, Kyoung-Bun Lee, Tae Jin Kim, Curie Ahn, Jaeseok Yang

**Affiliations:** 1 Department of Internal Medicine, Hallym University College of Medicine, 150 Seongan-ro, Gangdong-gu, Seoul, 05355 Republic of Korea; 2 Division of Clinical Bioinformatics, Biomedical Research Institute, Seoul National University Hospital, 101 Daehak-ro, Jongno-gu, Seoul, 03080 Republic of Korea; 3 Transplantation Research Institute, Seoul National University College of Medicine, 101 Daehak-ro, Jongno-gu, Seoul, 03080 Republic of Korea; 4 Department of Internal Medicine, Seoul National University College of Medicine, 101 Daehak-ro, Jongno-gu, Seoul, 03080 Republic of Korea; 5 Department of Nephrology, Ajou University School of Medicine, 164 Worldcup-ro, Yeongtong-gu, Suwon, 16499 Republic of Korea; 6 Department of Pathology, Seoul National University Hospital, 101 Daehak-ro, Jongno-gu, Seoul, 03080 Republic of Korea; 7 Division of Immunobiology, Sungkyunkwan University School of Medicine, 2066 Seobu-ro, Jangan-gu, Suwon, 16419 Republic of Korea; 8 Transplantation Center, Seoul National University Hospital, 101 Daehak-ro, Jongno-gu, Seoul, 03080 Republic of Korea; 9 Department of Surgery, Seoul National University Hospital, 101 Daehak-ro, Jongno-gu, Seoul, 03080 Republic of Korea

**Keywords:** ABO incompatible kidney transplantation, B cell receptor, RNA-seq, VDJ usage

## Abstract

**Background:**

Studies on B-cell subtypes and V(D)J gene usage of B-cell receptors in kidney transplants are scarce. This study aimed to investigate V(D)J gene segment usage in ABO-incompatible (ABOi) kidney transplant (KT) patients compared to that in ABO-compatible (ABOc) KT patients.

**Methods:**

We selected 16 ABOi KT patients with accommodation (ABOiA), 6 ABOc stable KT patients (ABOcS), and 6 ABOi KT patients with biopsy-proven acute antibody-mediated rejection (ABOiR) at day 10, whose graft tissue samples had been stored in the biorepository between 2010 and 2014. Complete transcriptomes of graft tissues were sequenced and analyzed through RNA sequencing (RNA-seq). The international ImMunoGeneTics information system (IMGT®) was used for in-depth comparison of V(D)J gene segment usage.

**Results:**

The mean age of the 28 KT recipients was 43.3 ± 12.8 years, and 53.6% were male. By family, IGHV3, IGHJ4, IGLV2, and IGLJ3 gene segments were most frequently used in all groups, and their usage was not statistically different among the three patient groups. While IGKV3 was most frequently used in both the ABOiA and ABOiR groups, IGKV1 was most commonly used in the ABOcS group. In addition, while IGKJ1 was most commonly used in the ABOiA and ABOcS groups, IGKJ4 was most frequently used in the ABOiR group. According to individual gene segments, IGHV4–34 and IGHV4–30-2 were more commonly used in the ABOiR group than in the ABOiA group, and IGHV6–1 was more commonly used in the ABOcS group than in the ABOiR group. IGLV7–43 was more commonly used in the ABOcS group than in the ABOi group. However, technical variability, small sample size, and potential confounding effects of Rituximab or HLA mismatching are limitations of our study.

**Conclusions:**

Our findings suggest that RNA-seq transcriptomic analyses can provide information on the V(D)J gene usage of B-cell receptors and the mechanisms of accommodation and immune reaction in ABOi KT.

## Background

The number of patients with end-stage renal disease requiring kidney transplantation (KT) is growing rapidly; however, donor organ shortages are becoming a major problem throughout the world [1]. ABO-incompatible (ABOi) KT is one solution to this problem and is known to increase organ transplantation by approximately 20%. Recently, development of both more potent immunosuppressive agents and desensitization methods has increased the number of ABOi KT and has improved its outcomes [2].

The presence of anti-ABO antibody against the donor blood group is the major immunological obstacle to successful ABOi KT. Pre-transplantation desensitization with anti-CD20 monoclonal antibody and plasmapheresis is performed to suppress the production of anti-ABO antibody and to remove pre-formed antibody. These desensitization therapies may prevent the occurrence of hyperacute and acute antibody-mediated rejection (AMR) [3]. However, anti-ABO antibody usually returns and persists after ABOi KT despite adequate immunosuppression [4]. Even with the continued presence of anti-ABO antibody in the patient’s serum and the presence of the target antigen in the donor kidney, anti-ABO antibody-mediated tissue injury may not occur and the graft can continue to function well; this phenomenon is termed accommodation [5]. However, the mechanism of accommodation between anti-ABO antibody and the kidney graft remains unclear. Moreover, there is a lack of studies on what kinds of B-cell subtypes play key roles in these immune responses, and studies regarding the variable (V), diversity (D), and joining (J) gene segment usages of B-cell receptors in ABOi KT are also scarce.

VDJ gene segment usage has been reported in several animal and human studies. Altered VDJ gene segment utilization in antibody response by aging stages were shown in mice and horse models [6–8]. In human, the analyses of VDJ gene segment usage were performed in immune response to microbial antigens including Haemophilus influenza and rabies [9–12]. Moreover, shared immunoglobulin heavy chain V1–46 gene usage by autoantibodies was identified in humoral immune reaction among patients with pemphigus vulgaris [13].

The aims of the current study were to investigate the VDJ gene segments that are mainly used in B-cell receptors in ABOi KT with accommodation or acute AMR compared to those used in ABO-compatible kidney transplantation (ABOc KT) through RNA sequencing (RNA-seq) transcriptomic analysis.

## Methods

The running of a biorepository of graft tissue samples of KT patients (H-1102-082-353) and this study (H-1412-029-631) were performed with approval from the Institutional Review Board of Seoul National University Hospital. This study was performed in accordance with the Helsinki Declaration of 2000 and the Declaration of Istanbul 2008.

### Patient and sample selection

We selected 16 ABOi KT recipients who showed accommodation (ABOiA) with neither rejection on protocol biopsy on day 10 nor abnormal renal function during the first 3 months after KT and whose graft tissue samples had been stored in the biorepository between June 2010 and August 2014. Six stable ABOc KT recipients (ABOcS) who had good graft function, stable creatinine levels, and normal 10th-day protocol biopsy findings were selected as the control group. Additionally, six ABOi KT recipients who had acute AMR (ABOiR) at their 10th-day protocol biopsy were also selected for comparison. At our institution, living-donor ABOi KT is performed with the following desensitization protocol: Rituximab, a chimeric monoclonal antibody for CD20, is administered at a single dose (125 mg/m^2^) within 7 days before initiation of plasmapheresis. Plasmapheresis is performed before kidney transplantation to remove pre-formed isoagglutinin (anti-ABO) antibodies. The number of rounds of plasmapheresis is determined according to the baseline anti-donor isoagglutinin IgG titer, and the acceptable isoagglutinin IgG titer before operation is 1:16 or less. Anti-ABO isoagglutinin antibody titers are measured by two-fold serial dilution of the recipient’s serum. A commercially available gel card test (Sigma-Aldrich, St. Louis, MO, USA) is utilized and is known to be more qualitative in grading the agglutination reaction than the tube test [14]. After each session of plasmapheresis, a low dose of intravenous immunoglobulin (100 mg/kg) is administered. All patients receive 20 mg basiliximab on days 0 and 4 as an induction therapy, and conventional triple maintenance immunosuppressive agents are administered, including tacrolimus, prednisolone, and mycophenolate mofetil.

### Sample preparation and RNA-seq

All graft tissue samples from kidney transplantation were stored at −70 °C until being processed for total RNA extraction. Total RNA was extracted from the graft tissue using the RNeasy® Mini kit (Qiagen, Valencia, CA, USA). RNAs released from the tissue were prepared into adapter-ligated complementary DNA (cDNA) libraries through fragmentation, reverse transcription, and amplification with random oligo-dT primers according to the Illumina TruSeq protocol. The cDNA libraries were sequenced using an Illumina HiSeq platform (Illumina Inc., San Diego, CA, USA). Illumina HiSeq generates raw images utilizing HiSeq Control Software v2.2.38 for system control (Illumina Inc.) and base calls through an integrated primary analysis software called Real Time Analysis. v1.18.61.0 (Illumina Inc.). The base call binary data were converted into FASTQ utilizing the Illumina package bcl2fastq (v1.8.4, Illumina Inc.). No target-specific amplification was used in our RNA-seq method.

### Immunoglobulin VDJ gene sequence analyses

The short reads obtained by RNA-seq underwent TruSeq adapter trimming using Trimmomatic [15]. Then, we aligned the adapter-trimmed short reads to the international ImMunoGeneTics (IMGT®, http://www.imgt.org) reference sequence using Burrows-Wheeler Aligner [16]. In this study, the IMGT® database was downloaded, and immunoglobulin sequence data for *Homo sapiens* were extracted to be used as a reference sequence [17]. The aligned output was sorted using Picard AddOrReplaceReadGroups (http://broadinstitute.github. io/picard). The number of reads aligned to each VDJ gene segment was counted using SAMtools [18]. Then, read counts aligned to VDJ gene segments of immunoglobulin heavy or light chains were compared among the three patient groups. The mean value of the mean depth-of-coverage of the immunoglobulin segments was 29.0 ± 8.6.

### Statistical analyses

Chi-square tests were used for categorical variables. A one-way analysis of variance test was used to compare continuous variables among the groups, and post-hoc analyses were also performed. All statistical analyses were conducted using SPSS statistical software (version 22.0; SPSS Inc., Chicago, IL, USA) and the software package R version 3.2.1 (The R Foundation for Statistical Computing, Vienna, Austria; www.r-project.org). A *P*-value of <0.05 was considered statistically significant.

## Results

### Demographics and clinical characteristics of the subjects

The mean age at KT was 43.3 ± 12.8 (standard deviation) years, and 53.6% of the 28 KT recipients were male. The most common cause of end-stage renal disease before KT was glomerulonephritis, followed by polycystic kidney disease, diabetes mellitus, and hypertension. Three (10.7%) patients had diabetes, and 25 (89.3%) patients had hypertension. In ABOi KT, the most common ABO incompatibility was ‘B to A’ or ‘B to O.’ All of the recipients received tacrolimus as a calcineurin inhibitor except for one patient in the ABOc KT group. Two patients were positive for hepatitis B surface antigen, and one patient was positive for anti-hepatitis C virus antibody. The mean age of donors at KT was 43.0 ± 10.7 years, and 57.1% were male. The majority of parameters were not statistically different among the three groups, with the exception being the number of human leukocyte antigen (HLA) mismatches. However, none of the recipients had donor-specific anti-HLA antibodies at the time of the 10th-day protocol biopsy. A comparison of clinical characteristics among the three groups is presented in Table 1.

**Table 1:** Clinical characteristics of ABO-incompatible accommodation, ABO-compatible stable, and ABO-incompatible rejection kidney transplantation groups

Parameters	ABO-incompatible accommodation group (*n* = 16)	ABO-compatible stable group (*n* = 6)	ABO-incompatible rejection group (n = 6)	*P*-value^a^
Age at transplantation (yr)	40.8 ± 13.8	44.2 ± 11.7	49.2 ± 11.1	0.406
Male sex (%)	9 (56.2%)	2 (33.3%)	4 (66.7%)	0.485
Body mass index (kg/m^2^)	21.6 ± 2.3	20.5 ± 3.0	22.9 ± 1.9	0.242
Diabetes (%)	2 (12.5%)	0 (0.0%)	1 (16.7%)	0.608
Hypertension (%)	13 (81.2%)	6 (100.0%)	6 (100.0%)	0.284
ABO incompatibility (%)				<0.001
- A to B	0 (0.0%)		1 (16.7%)	
- A to O	4 (25.0%)		1 (16.7%)	
- AB to A	1 (6.2%)		0 (0.0%)	
- AB to B	3 (18.8%)		0 (0.0%)	
- B to A	5 (31.2%)		1 (16.7%)	
- B to O	3 (18.8%)		3 (50.0%)	
Pre-transplant dialysis (%)				0.122
- Hemodialysis	12 (75.0%)	4 (66.7%)	3 (50.0%)	
- Peritoneal dialysis	0 (0.0%)	2 (33.3%)	2 (33.3%)	
- Preemptive	4 (25.0%)	0 (0.0%)	1 (16.7%)	
Duration of dialysis (mo)	30.7 ± 58.1	24.2 ± 52.9	8.5 ± 12.5	0.667
Cause of ESRD (%)				0.590
- Diabetes	2 (12.5%)	0 (0.0%)	1 (16.7%)	
- Glomerulonephritis	10 (62.5%)	4 (66.7%)	3 (50.0%)	
- Hypertension	0 (0.0%)	1 (16.7%)	0 (0.0%)	
- Polycystic disease	3 (18.8%)	0 (0.0%)	1 (16.7%)	
- Unknown	1 (6.2%)	1 (16.7%)	1 (16.7%)	
Serum creatinine at discharge (mg/dL)	1.1 ± 0.2	0.9 ± 0.1	1.5 ± 1.3	0.186
Estimated GFR at discharge (mL/min)	86.8 ± 24.5	95.9 ± 13.9	74.2 ± 32.8	0.328
Serum creatinine at 3 years after transplantation (mg/dL)	1.7 ± 1.7	1.1 ± 0.1	1.2 ± 0.5	0.651
Estimated GFR at 3 years after transplantation (mL/min)	54.6 ± 19.4	57.0 ± 7.2	59.1 ± 15.7	0.878
Calcineurin inhibitor (%)				0.149
- Tacrolimus	16 (100.0%)	5 (83.3%)	6 (100.0%)	
- Cyclosporine	0 (0.0%)	1 (16.7%)	0 (0.0%)	
Trough level of tacrolimus at discharge (ng/mL)	9.3 ± 2.3	9.1 ± 1.0	10.8 ± 3.0	0.382
HLA mismatch (number)	2.9 ± 1.3	5.2 ± 0.8	2.8 ± 1.2	0.001
HBsAg positivity (%)	1 (6.2%)	1 (16.7%)	0 (0.0%)	0.522
Anti-HCV positivity (%)	0 (0.0%)	1 (16.7%)	0 (0.0%)	0.149
Donor				
- Age at transplantation (yr)	43.9 ± 10.9	43.5 ± 7.5	40.3 ± 14.0	0.795
- Male sex (%)	7 (43.8%)	5 (83.3%)	4 (66.7%)	0.215
- Body mass index (kg/m^2^)	23.3 ± 2.9	23.3 ± 2.0	24.8 ± 2.2	0.437
- Serum creatinine (mg/dL)	0.8 ± 0.2	0.8 ± 0.1	0.9 ± 0.2	0.272

*ESRD* end-stage renal disease, *GFR* glomerular filtration rate, *HLA* human leukocyte antigen, *HBsAg* hepatitis B surface antigen, *HCV* hepatitis C virus, *mo* months

Numerical values are expressed as mean ± standard deviation, and categorical values are expressed as frequency (percentage)

^a^Continuous variables were compared using one-way analysis of variance, and categorical variables were compared using the chi-squared test, as appropriate

In addition, CD20 immunohistochemistry staining for determining the infiltration of B cells in the kidney allograft tissue samples was performed in our study patients. B cell infiltration in kidney tissue were observed, and more increased grade of B cell infiltration in ABOiR KT was identified than ABOiA KT.

### Immunoglobulin heavy chain gene segment usage

**Fig. 1:**
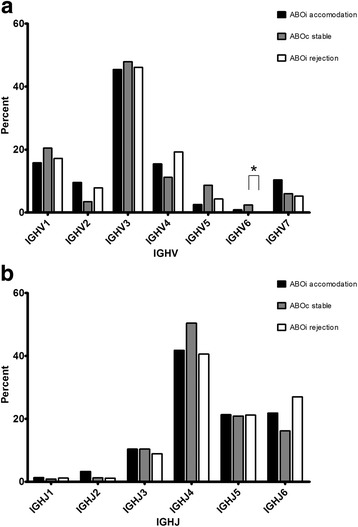
Immunoglobulin heavy chain V and J gene segment family usage in renal allograft tissue transcripts. Percent of unique, in-frame sequences using the indicated V (**a**) and J (**b**) gene segment families in ABO-incompatible (ABOi) accommodation, ABO-compatible (ABOc) stable, and ABOi rejection groups after kidney transplantation. All comparisons were performed with one-way analysis of variance and post-hoc analyses. IGHV, immunoglobulin heavy chain variable; IGHJ, immunoglobulin heavy chain joining. * *P* < 0.05

**Fig. 2:**
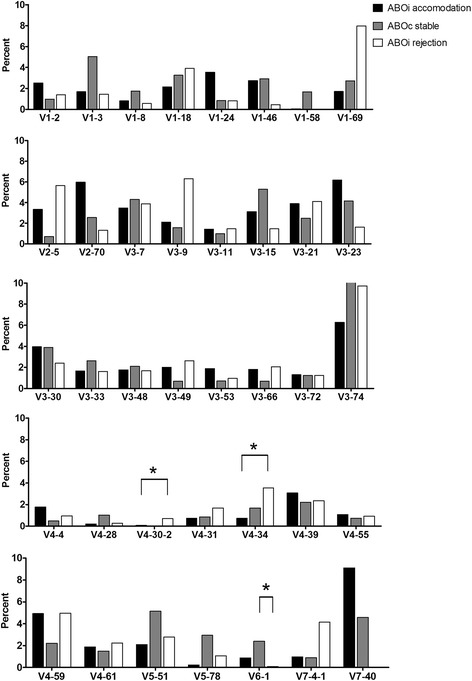
Immunoglobulin heavy chain V gene segment usage in renal allograft tissue transcripts. Percent of unique, in-frame sequences using the indicated gene segment families in ABO-incompatible (ABOi) accommodation, ABO-compatible (ABOc) stable, and ABOi rejection groups after kidney transplantation. All comparisons were performed with one-way analysis of variance and post-hoc analyses. * *P* < 0.05

In the ABOiA group, immunoglobulin heavy chain V domain 3 (IGHV3) gene segments were most frequently used, followed by those in the IGHV1, IGHV4, IGHV7, IGHV2, IGHV5, and IGHV6 heavy chain V families (Fig 1a). According to individual V gene segments, IGHV7–40, IGHV3–74, IGHV3–23, and IGHV2–70 were most common in the ABOiA group (Fig 2). In the ABOcS group, IGHV3 gene segments were most commonly used, followed by IGHV1, IGHV4, IGHV5, IGHV7, IGHV2, and IGHV6 in the heavy chain V family (Fig 1a). Among individual V gene segments, IGHV3–74, IGHV1–3, IGHV3–15, IGHV5–51, and IGHV7–40 were the most common in the ABOcS group (Fig 2). In the ABOiR group, IGHV3 gene segments were most frequently used, followed by IGHV4, IGHV1, IGHV2, IGHV7, IGHV5, and IGHV6 in the heavy chain V family (Fig 1a). Among individual V gene segments, IGHV3–74, IGHV1–69, IGHV3–9, IGHV2–5, and IGHV4–59 were the most common in the ABOiR group (Fig 2). By family, the frequencies of heavy chain V gene segment usage were not different among the three groups except for that of IGHV6. The ABOcS group was enriched for IGHV6 usage compared to that in the ABOiR group (Fig 1a). According to analysis of individual heavy chain V gene segments, IGHV4–30-2 and IGHV4–34 were more common in the ABOiR group than in the ABOiA group, and IGHV6–1 was more common in the ABOcS group than in the ABOiR group (Fig 2).

In the ABOiA group, immunoglobulin heavy chain J domain 4 (IGHJ4) gene segments were predominant, followed by IGHJ6, IGHJ5, IGHJ3, IGHJ2, and IGHJ1 in the heavy chain J family (Fig 1b). In the ABOcS group, IGHJ4 gene segments were also most commonly used, followed by IGHJ5, IGHJ6, IGHJ3, IGHJ2, and IGHJ1 (Fig 1b). Similarly, in the ABOiR group, IGHJ4 gene segments were most commonly used, followed by IGHJ6, IGHJ5, IGHJ3, IGHJ1, and IGHJ2 (Fig 1b). Among the three groups, the frequencies of heavy chain J gene segment usage were not statistically different.

### Immunoglobulin light kappa chain gene segment usage

**Fig. 3:**
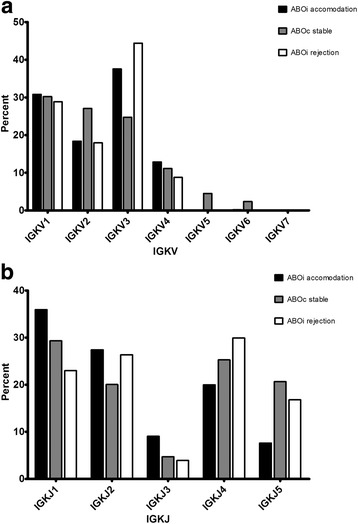
Immunoglobulin light kappa chain V and J gene family usage in renal allograft tissue transcripts. Percent of unique, in-frame sequences using the indicated V (**a**) and J (**b**) gene segments in ABO-incompatible (ABOi) accommodation, ABO-compatible (ABOc) stable, and ABOi rejection groups after kidney transplantation. All comparisons were performed with one-way analysis of variance and post-hoc analyses. IGKV, immunoglobulin light kappa chain variable; IGKJ, immunoglobulin light kappa chain joining. * *P* < 0.05

**Fig. 4:**
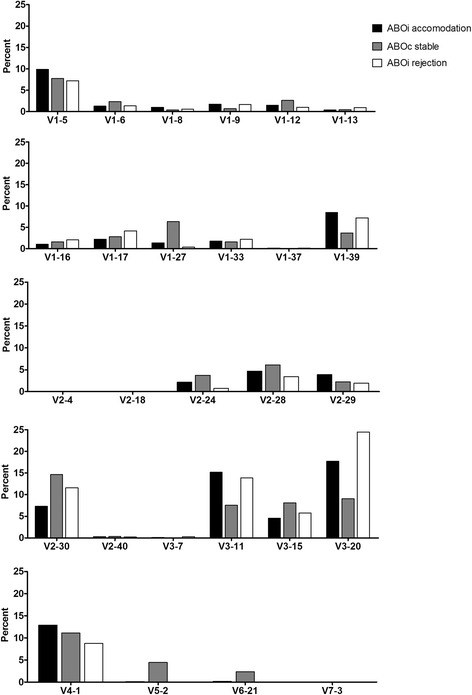
Immunoglobulin light kappa chain V gene segment usage in renal allograft tissue transcripts. Percent of unique, in-frame sequences using the indicated gene segments in ABO-incompatible (ABOi) accommodation, ABO-compatible (ABOc) stable, and ABOi rejection groups after kidney transplantation. All comparisons were performed with one-way analysis of variance and post-hoc analyses. * *P* < 0.05

In the ABOiA group, immunoglobulin light kappa chain V domain 3 (IGKV3) gene segments were most frequently used, followed by IGKV1, IGKV2, IGKV4, IGKV6, IGKV5, and IGKV7 in the light kappa chain V family (Fig 3a). In terms of individual V gene segments, IGKV3–20, IGKV3–11, IGKV4–1, IGKV1–5, and IGKV1–39 were the most common in the ABOiA group (Fig 4). In the ABOcS group, IGKV1 gene segments were most commonly used, followed by IGKV2, IGKV3, IGKV4, IGKV5, and IGKV6 in the light kappa chain V family (Fig 3a). The individual V gene segments IGKV2–30, IGKV 4–1, IGKV3–20, IGKV3–15, IGKV1–5, and IGKV3–11 were the most common in the ABOcS group (Fig 4). In the ABOiR group, IGKV3 gene segments were most frequently used, followed by IGKV1, IGKV2, and IGHV4 in the light kappa chain V family (Fig 3a). The individual V gene segments IGKV3–20, IGKV3–11, IGKV2–30, IGKV4–1, IGKV1–39, and IGKV1–5 were the most common in the ABOiR group (Fig 4). By family, frequencies of light kappa chain V gene segment usage did not statistically differ among the three groups. However, while the IGKV3 gene was the most commonly used in both the ABOiA and ABOiR groups, the IGKV1 gene was most frequently used only in the ABOcS group (Fig 3a). In terms of individual light kappa chain V gene segments, frequencies of gene segment usage were not statistically different among the three groups (Fig 4).

In the ABOiA group, immunoglobulin light kappa chain J domain 1 (IGKJ1) gene segments were predominant, followed by IGKJ2, IGKJ4, IGKJ3, and IGKJ5 in the light kappa chain J family (Fig 3b). In the ABOcS group, IGKJ1 gene segments were most commonly used, followed by IGKJ4, IGKJ5, IGKJ2, and IGKJ3 in the light kappa chain J family (Fig 3b). In the ABOiR group, IGKJ4 gene segments were most commonly used, followed by IGKJ2, IGKJ1, IGKJ5, and IGKJ3 (Fig 3b). However, there were no differences in the frequencies of light kappa chain J gene segment usage among the three groups.

### Immunoglobulin light lambda chain gene segment usage

**Fig. 5:**
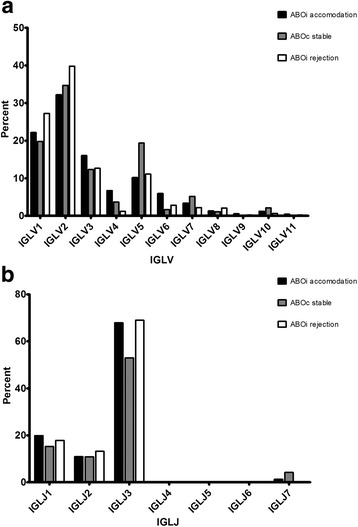
Immunoglobulin light lambda chain V and J gene family usage renal allograft tissue transcripts. Percent of unique, in-frame sequences using the indicated V (**a**) and J (**b**) gene segments in ABO-incompatible (ABOi) accommodation, ABO-compatible (ABOc) stable, and ABOi rejection groups after kidney transplantation. All comparisons were performed with one-way analysis of variance and post-hoc analyses. IGLV, immunoglobulin light lambda chain variable; IGLJ, immunoglobulin light lambda chain joining. * *P* < 0.05

**Fig. 6:**
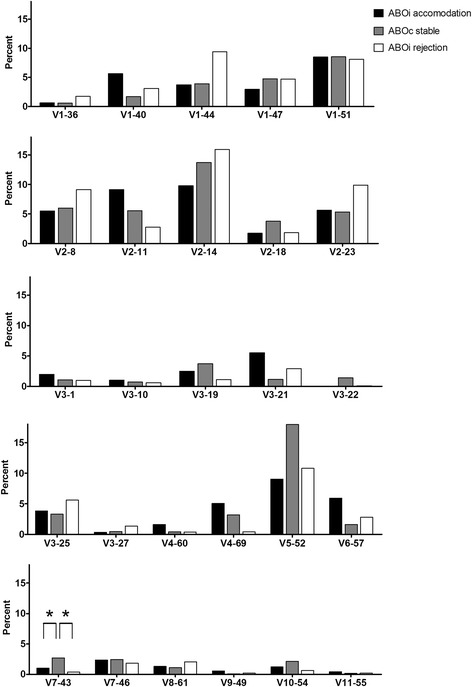
Immunoglobulin light lambda chain V gene segment usage in renal allograft tissue transcripts. Percent of unique, in-frame sequences using the indicated gene segment in ABO-incompatible (ABOi) accommodation, ABO-compatible (ABOc) stable, and ABOi rejection groups after kidney transplantation. All comparisons were performed with one-way analysis of variance and post-hoc analyses. * *P* < 0.05

In the ABOiA group, immunoglobulin light lambda chain V domain 2 (IGLV2) gene segments were the most frequently used, followed by the IGLV1, IGLV3, IGLV5, IGLV4, IGLV6, IGLV7, IGLV8, IGLV10, IGLV9, and IGLV11 light lambda chain V family members (Fig 5a). Among individual V gene segments, IGLV2–14, IGLV2–11, IGLV5–52, and IGLV1–51 were the most common in the ABOiA group (Fig 6). In the ABOcS group, IGLV2 gene segments were most common, followed by IGLV1, IGLV5, IGLV3, IGLV7, IGLV4, IGLV10, IGLV6, IGLV8, IGLV11, and IGLV9 in the light lambda chain V family (Fig 5a). Among individual V gene segments, IGLV5–52, IGLV2–14, IGLV1–51, IGLV2–8, IGLV2–11, and IGLV2–23 were most frequently used in the ABOcS group (Fig 6). In the ABOiR group, IGLV2 gene segments were most frequently used, followed by IGLV1, IGLV3, IGLV5, IGLV6, IGLV7, IGLV8, IGLV4, IGLV10, IGLV11, and IGLV9 in the light lambda chain V family (Fig 5a). In terms of individual V gene segments, IGLV2–14, IGLV5–52, IGLV2–23, IGLV1–44, IGLV2–8, and IGLV1–51 were most common in the ABOiR group (Fig 6). By family, frequencies of light lambda chain V gene segment usage were not statistically different among the three groups (Fig 5a). Similarly, based on individual light lambda chain V gene segments, frequencies of gene segment usage were not statistically different among the three groups, except for in the case of IGLV7–43, which was more commonly used in the ABOcS group than in both the ABOiA and ABOiR groups (Fig 6).

In the ABOiA group, immunoglobulin light lambda chain J domain 3 (IGLJ3) gene segments were predominant, followed by the IGLJ1, IGLJ2, IGLJ7, and IGLJ6 light lambda chain J family members (Fig 5b). In the ABOcS group, IGLJ3 gene segments were most commonly used, followed by IGLJ1, IGLJ2, and IGLJ7 (Fig 5b). In the ABOiR group, IGLJ3 gene segments were most commonly used, followed by IGLJ1 and IGLJ2 (Fig 5b). Among the three groups, the frequencies of light lambda chain J gene segment usage were not statistically different.

## Discussion

The present study used RNA-seq transcriptomic analysis to compare the V(D)J gene segment usage of B-cell receptors in ABOi and ABOc KT recipients. By family, the IGHV3 and IGHJ4 gene segments were the most frequently used in all groups, and usage did not statistically differ among the three groups. In terms of individual heavy chain V gene segments, IGHV4–34 and IGHV4–30-2 were more often used in the ABOiR group than in the ABOiA group, and IGHV6–1 was more often used in the ABOcS group than in the ABOiR group. While the IGKV3 gene segment family was most frequently used in both the ABOiA and ABOiR groups, the IGKV1 gene segment was most commonly used in the ABOcS group. The IGKJ1 gene segment was most commonly used in the ABOiA and ABOcS groups, and the IGKJ4 gene segment was most frequently used in the ABOiR group. By family, the IGLV2 and IGLJ3 gene segments were the most frequently used in all groups, and rates of usage were not statistically different among the three groups. IGLV7–43 was used more often in the ABOcS group than in both the ABOiA and ABOiR groups.

Mroczek et al. reported an in-depth comparison of V(D)J gene usage among heavy chain repertoires expressed by each B-cell subset isolated from the blood of a single healthy individual [19]. B-cell subsets in this study included immature, transitional, mature, IgD^+^ memory, IgD^−^ memory, and plasma cells. Their results indicated that heavy chain repertoires and V(D)J gene usages differed in terms of sequence content among different B-cell subsets. In their study, plasma cells exhibited a higher rate of usage of IGHV4–34 than that of other B-cell subsets, and this contributed to a higher usage of the IGHV4 heavy chain V family. In our study, IGHV4–34 was more frequently used in the ABOiR group than in the ABOiA group. These results suggest that plasma cells may have a critical role in acute AMR in the early period after ABOi KT. Cases of acute AMR in the present study are believed to occur mainly with anti-ABO isoagglutinin antibodies, as no recipients in the ABOiR group had donor specific anti-HLA antibodies at the time of the 10th-day protocol biopsy. Recently, intensive desensitization therapy has decreased the incidence of AMR in ABOi KT and improved its outcomes [20]. However, some patients still experience AMR due to under-immunosuppression, while other patients suffer complications due to over-immunosuppression. Therefore, it is important to understand the mechanisms of accommodation more clearly and to develop biomarkers capable of differentiating AMR from accommodation in ABOi KT for individualized immunosuppression. Our results suggest that the IGHV4–34 gene segment may be a promising biomarker for acute AMR in the early period after ABOi KT.

Next, we identified differences in the usage of several gene segments between ABOi KT and ABOc KT. Usage of the IGKV3 and IGKV1 gene segment families and of the IGLV7–43 gene segment were significantly different between these groups of patients. Pre-transplant desensitization therapy, including anti-CD20 depleting antibody and plasmapheresis, may have led to different patterns of gene segment usage in B cells in ABOi KT compared to that in ABOc KT without desensitization. Differences between ABOc KT and ABOi KT patients may be secondary to rituximab therapy which depletes the patient’s original B cell repertoire and replace it with a completely repertoire as the B cell count recover. However, these results may also be attributed to differing roles of B-cell subsets in the antibody response between ABOi KT and ABOc KT. B-1 cells are a subtype of B cells that express self-reactive B-cell receptor and produce a significant portion of IgM natural antibodies [21]. B-2 cells make up the majority of B cells in the spleen and lymph nodes and display high levels of IgD. However, B-1 cells that are enriched in the peritoneal and pleural cavities in the adult animal [22, 23] comprise less than 5% of B cells in the spleen and express lower levels of IgD. Although the presence and role of human B-1 cells have not yet been clarified, it has been suggested that human B-1 cells may play a role similar to that of murine B-1 cells. Griffin et al. reported the presence of human B-1 cells in adult peripheral blood using the surface marker profile CD20^+^CD27^+^CD43^+^CD70^−^ [24]. Anti-ABO antibody is a natural human antibody and is reported to be produced by B-1 cells [25]. While conventional B-2 cells produce antibodies against peptide antigens, B-1 cells produce antibodies against carbohydrate antigens such as anti-ABO antibodies. The majority of B-1 cells express B-cell receptors composed of IGHV11, IGHJ1, and IGKV9 gene segments in adult mouse [26]; however, it is not known which VDJ gene segments are most commonly used in human B-1 cells. Taken together, the differences in VDJ usage between ABOi and ABOc KT may be explained by the dominant role of B-1 cells or another B-cell subset that plays an important role in the anti-ABO antibody response. For elucidation of this issue, further studies on VDJ usage in human B-1 cells and marginal zone B cells will be required [19].

Mroczek et al. showed that IGVH4 was the most frequently used gene segment family in all types of B-cell subsets [19]. However, in the present study, IGVH3 was the most commonly used gene segment family in all KT groups regardless of ABO compatibility. Moreover, the read counts for immunoglobulin heavy chain D gene usage were too low to analyze differences between groups. These results suggest that KT itself or conventional triple immunosuppression including steroid, calcineurin inhibitor, and anti-metabolite may affect the usage of immunoglobulin heavy chain V and D gene segments in B cells compared to that in healthy controls. However, the predominance of IGHJ4 and the frequencies of immunoglobulin heavy chain J gene segment usage appear to be similar between the two studies.

Recently, concept of iWAS (immunonome Wide Association Study) was proposed and the regression can be performed by regressing on each T cell or B cell receptor marker individually, as in a classical GWAS [27]. In the present study, we have already presented differences in the VDJ sequence usage among ABOi KT with accommodation, ABOi KT with rejection, and stable ABOc KT as one of efforts to correlate BCR repertoire with immunological phenotypes. When we applied iWAS approach using regression analysis to determine association of individual BCR usage with ABO incompatibility in KT patients, we could not make a good predictive model (data not shown). To use iWAS approach better, future studies are warranted to analyze BCR usage in sorted B cell subsets that are eluted from graft tissues or are derived from peripheral blood.

The present study has some limitations. First, we did not sort B cells or immune cells before analysis of B-cell receptors, and the entire graft tissue was used for RNA-seq. Sorting of B cells or immune cells may provide better qualitative results; however, RNA-seq data using the entire graft tissue is also reliable because RNA-seq transcriptome sequencing has a high specificity and low false-positive rate, i.e. no background signals originate from cross-hybridization, which occurs with microarrays. Second, we were not able to confirm the potential biomarkers we identified, such as IGHV4–34, which were differentially expressed among KT groups, by real-time polymerase chain reaction owing to the lack of sufficient quantities of graft tissue samples. Lack of external validation samples was another limitation of this study. Third, differences in the VDJ usage might have been affected from differences in HLA matching or anti-HLA antibody responses among different patients rather than determined by ABO compatibility or presence of rejection. Technical variability in RNA-seq, small sample size, and confounding effects of B cell depletion by Rituximab are also potential limitations. Further, larger-scaled studies are warranted to confirm the results of our study Nevertheless, the present study is the first to investigate the VDJ gene segment usage of B-cell receptors in kidney allografts through RNA-seq transcriptomic analysis.

## Conclusions

Our findings suggest that RNA-seq transcriptomic analyses can provide information on the V(D)J gene usages of B-cell receptors and the mechanisms of accommodation and immune reactions in ABOi KT. These V(D)J gene usage patterns may reveal potential candidates for biomarkers to detect accommodation in ABOi KT.

## Abbreviations

ABOc: ABO-compatible
ABOcS: ABO-compatible stable
ABOi: ABO-incompatible
ABOiA: ABO-incompatible accommodation
ABOiR: ABO-incompatible rejection
AMR: Antibody-mediated rejection
cDNA: Complementary DNA
D: Diversity
HLA: Human leukocyte antigen
IGHJ: Immunoglobulin heavy chain joining
IGHV: Immunoglobulin heavy chain variable
IGKJ: Immunoglobulin light kappa chain joining
IGKV: Immunoglobulin light kappa chain variable
IGLJ: Immunoglobulin light lambda chain joining
IGLV: Immunoglobulin light lambda chain variable
J: Joining
KT: Kidney transplantation
RNA-seq: RNA sequencing
V: Variable
